# Stratification of gastric cancer risk based on family history with cost-effectiveness of opportunistic endoscopic screening: a hospital-based cross-sectional study (SIGES)

**DOI:** 10.3389/fonc.2026.1902158

**Published:** 2026-10-02

**Authors:** Zhuo-Yu Li, Wen Xiang, Yan Huang, Xin-Zu Chen

**Affiliations:** 1 Gastric Cancer Center, Department of General Surgery, West China Hospital, Sichuan University, Chengdu, China; 2 Department of Thyroid and Breast Surgery, Chengdu Fifth People’s Hospital, Chengdu, China; 3 Health Management Center, General Practice Medical Center, West China Hospital, Sichuan University, Chengdu, China; 4 Department of General Practice (Premium Medical Center/International Medical Center), West China Hospital, Sichuan University, Chengdu, China

**Keywords:** CA19-9, CEA, endoscopy, family history, gastric cancer, guideline, screening, serology

## Abstract

**Objectives:**

The strategy for opportunistic endoscopic screening driven by family history of gastric cancer needs to be optimized in a tailored manner through further risk stratification.

**Methods:**

A hospital-based cross-sectional study included health check-up controls and gastric cancer patients at West China Hospital, Sichuan University. Demographic characteristics, comorbidities, family history of malignancies, and serology were analyzed for associations with gastric cancer risk. Associations between family history of gastric cancer and incident gastric cancer risk were assessed, and a rapid cost-effectiveness analysis was performed.

**Results:**

In total, 58,218 observations were analyzed, including 619 gastric cancer patients. Family history of gastric cancer was an independent risk factor for gastric cancer (adjusted odds ratio [aOR] = 5.59, 95% CI 3.81–8.20) and was associated with an increased risk of upper digestive tract benign comorbidities (aOR = 1.51, 95% CI 1.09–2.71). For persons with a family history of gastric cancer who were older than 40 years (odds ratio [OR] = 2.36, 95% CI 1.29–4.32), endoscopic screening could be mildly recommended in general practice. Endoscopic screening should be moderately recommended for those with a family history of gastric cancer if they were older than 60 years (OR = 8.90, 95% CI 3.84–20.61) and strongly recommended if they also had upper digestive tract benign comorbidities (OR = 16.78, 95% CI 7.03–40.06), seropositivity for carcinoembryonic antigen (CEA) and/or carbohydrate antigen 19-9 (CA19-9) (OR = 20.77, 95% CI 9.15–47.14), or anemia (OR = 80.11, 95% CI 33.07–194.05). A rapid cost-effectiveness analysis indicated that moderate-to-strong recommendations were preferred, but based on willingness-to-pay considerations, mild-to-strong recommendations had a considerable detection rate for gastric cancer. Sensitivity analysis yielded consistent findings for decision-making.

**Conclusions:**

Family history of gastric cancer is an independent risk factor for gastric cancer. Optimized risk stratification based on family history can improve the cost-effectiveness of detecting gastric cancer. The mechanism by which a family history of gastric cancer increases gastric cancer risk and a more tailored family history-driven endoscopic screening strategy require further investigation.

## Introduction

Gastric cancer is the fifth most common cancer and the fourth leading cause of cancer-related deaths in the world (1), with around 75% of newly diagnosed cases occurring in Asian countries, especially China, Japan, and Korea (2, 3). In China, gastric cancer has remained a heavy health burden for decades, due to relatively high mortality (4, 5). To date, the poor nationwide prognosis of gastric cancer is largely attributable to the low proportion of early-stage disease, generally no more than 20% and especially low at the subnational level (6, 7). Japan and Korea have implemented national mass screening programs for gastric cancer via endoscopy since 1983 and 1999, respectively, leading to higher early diagnosis rates and reduced mortality (3). However, organized mass screening for gastric cancer has not been widely established in China (8). Therefore, opportunistic endoscopic screening still requires further attention to raise the detection rate of gastric cancer (9, 10). Given the resource-intensive nature of endoscopy, a more precise selection strategy for endoscopic screening and surveillance is required to efficiently aid in risk classification and early detection (11).

Family history of gastric cancer is one of the strongest and most consistently reported non-modifiable risk factors for gastric cancer (12–14). Family history of gastric cancer is associated with improved early detection and therefore may improve survival after curative-intent surgery even in patients with advanced gastric cancer (15). Family history of gastric cancer has not been uniformly recommended for intensified endoscopic screening and surveillance for asymptomatic relatives, largely because of the lack of standardized risk stratification and cost-effectiveness evaluation. However, in the recent guidelines by the National Health Commission of the People’s Republic of China and relevant collaborations, family history of gastric cancer among first-degree relatives such as parents or children and second-degree relatives such as siblings is defined as one of the high-risk factors for gastric cancer for endoscopic screening among adults over 40–45 years old (16, 17). This provides the rationale for the present study to include second-degree relatives as an innovative component. Therefore, optimization of an endoscopic screening protocol that incorporates precise family history-driven criteria is needed to improve early detection while conserving limited endoscopic resources. The present study hypothesized that the selection strategy for family history-driven endoscopic screening candidates could be enhanced in a tailored manner by combining family history of gastric cancer with other risk factors for gastric cancer.

## Methods

### Study design

This was a high-volume hospital-based cross-sectional study within the **Si**chuan **G**astric Cancer **E**arly Detection and **S**creening (SIGES) research project. This study was based on a retrospective dataset collected between May 2016 and December 2016 at West China Hospital, Sichuan University (10). The observations included both healthy check-up controls and gastric cancer patients, who were simultaneously managed or treated.

### Ethics

The present study was approved by the Biomedical Ethics Committee of West China Hospital, Sichuan University (identifier: 2018-216-V1, Aug 6, 2018). This study retrospectively collected the observations’ electronic medical records. Therefore, informed consent was waived by the Biomedical Ethics Committee of West China Hospital due to the retrospective nature of the study, while the personal information was anonymized during the whole procedure of analyzing and reporting data.

### Eligibility

For gastric cancer patients, the inclusion criteria were (1) the first diagnosis coded as ICD-10 C16 in the electronic medical record system; (2) age ≥18 years; and (3) incident gastric cancer confirmed by endoscopy and biopsy regardless of tumor-node-metastasis (TNM) stage, while the exclusion criteria were (1) other types of malignancies, such as lymphoma or gastrointestinal stromal tumor; (2) lack of pathological confirmation; (3) gastric cancer that was not newly diagnosed; and (4) incomplete data. For healthy check-up controls, the inclusion criteria were (1) selection from the health check-up registry system; (2) age ≥18 years; and (3) tumor-free status as reported by the general practitioners, while the exclusion criteria were (1) any suspected tumor; (2) self-reported comorbidities including the precancerous conditions of atrophic gastritis, intestinal metaplasia, and intraepithelial neoplasia; and (3) incomplete data.

### Data collection

The data were accessed from the electronic medical record system in April 2022. The retrieved information included questionnaire and serology data. The questionnaire included demographic features comprising sex (female, male) and age (≤30, 31–40, 41–50, 51–60, 61–70, >70 years), family history namely having a malignancy proband (the first individual diagnosed with cancer in a family) among first- or second-degree relatives (none, any malignancy other than gastric cancer, gastric cancer), and self-reported comorbidities (none, any non-digestive benign disease, previous or current upper digestive tract benign diseases including *Helicobacter pylori* infection, chronic gastritis, peptic ulcer, polyp, reflux, and dyspepsia). The questionnaire items were classified as specified above. The serology data included hemoglobin (HGB), carcinoembryonic antigen (CEA), and carbohydrate antigen 19-9 (CA19-9) (18, 19). Anemia was classified as none (female HGB ≥110 g/L, male HGB ≥120 g/L), mild (HGB lower than the cutoff but ≥90 g/L), and moderate-to-severe (HGB <90 g/L). Seropositivity of CEA and CA19-9 was identified using the in-house cutoffs of ≥5 ng/mL and ≥30 U/mL, respectively. The serologic tests were performed at the cross-sectional time point, either at the health check-up or at the diagnosis of gastric cancer. Additionally, the serologic multiplex of tumor biomarkers was stratified as CEA–/CA19-9–, CEA–/CA19-9+, CEA+/CA19-9–, and CEA+/CA19-9+.

### Outcomes and risk stratification

The primary outcome was the risk of incident gastric cancer. The secondary outcome was the identification of risk factors associated with a family history of gastric cancer. Subgroups included the family history of gastric cancer group, the family history of other malignancies group, and the no-family-history-of-malignancies group. In a decision-making model, a stepped sequential stratification of gastric cancer risk was considered by combining family history of gastric cancer with potential risk factors for gastric cancer, including sex, age, comorbidity, serum tumor biomarkers, and anemia.

### Statistics

Baseline characteristics of the observations were compared using chi-square test for categorical variables and the Wilcoxon rank-sum test for ranked variables or continuous variables. For the analysis of risk associations, both univariate and multivariate analyses were performed using logistic regression, and odds ratios (ORs) and adjusted odds ratios (aORs) with 95% confidence intervals (CIs) were estimated. All covariates were included in the regression models. Bootstrap resampling and estimation were performed with 200 repetitions in multivariate logistic regression. In the various sequential stratifications, the detection rate of gastric cancer was calculated (detection rate = detected cases/all observations × %), and the number needed to screen [NNS = 1/(detection rate of observational subgroup – detection rate of negative control group)] was estimated. A two-sided p-value < 0.05 was considered statistically significant. The STATA/SE 14.0 for Windows software was used for statistical analysis.

Additionally, a nested rapid health economic assessment was conducted to evaluate the strength of the recommendations (9, 10). The cost-effectiveness ratio (CER) and incremental cost-effectiveness ratio (ICER) were calculated based on the total direct medical cost and detection rate of each family history-driven endoscopic screening protocol. Protocol 1 (P1) included all family history-driven candidates for endoscopic screening without demographic-serologic stratification. In addition, demographic-serologic stratification was performed before sequential endoscopic screening in protocols 2, 3, and 4. Protocol 2 (P2) included family history-driven candidates with mild or stronger recommendations for endoscopic screening. Protocol 3 (P3) included family history-driven candidates with moderate or stronger recommendations for endoscopic screening. Protocol 4 (P4) included family history-driven candidates with strong recommendations for endoscopic screening. The total direct medical cost of each protocol was calculated as the sum of the costs of endoscopy [583.00 Chinese yuan (CNY)] and serology (CNY 15.00, CNY 33.00, and CNY 50.00 for blood cell count, serum CEA, and serum CA19–9 tests, respectively). Indirect medical or non-medical costs were not available in the present study. The false-positive rate (FPR) and false-negative rate (FNR) were calculated. A sensitivity analysis of CERs was performed by adjusting the number of healthy check-up controls from 1-fold (gastric cancer proportion: 1.1 per 100 persons) to 100-fold (gastric cancer proportion: 10.7 per 100,000 persons) among the proposed protocols.

### Registration and reporting

The protocol was registered elsewhere (ClinicalTrials.gov, identifier: NCT03597672). This cross-sectional study was reported according to the Strengthening the Reporting of Observational Studies in Epidemiology (STROBE) statement (20) and the REporting of studies Conducted using Observational routinely collected data (RECORD) (21).

## Results

### Baseline features

In total, 58,218 observations were collected in this cross-sectional study, including 619 (1.1%) gastric cancer patients. When comparing those with a family history of gastric or non-gastric malignancies with those with no family history of malignancies, the baseline characteristics were significantly different among various features, including sex, age, and comorbidity, except anemia (**Table 1**). The proportions of those with a family history of gastric cancer were 1.41% and 6.30% in healthy check-up controls and gastric cancer patients, respectively (p < 0.001) (**Table 1**). The seropositivity of CEA and CA19-9 and the serologic multiplex were comparable among family history of malignancy groups (**Table 2**).

**Table 1:** Baseline features of the present cross-sectional study.

Features	Family history of malignancies							
	None		Other malignancies			Gastric cancer		
	n	%	n	%	P_1_	n	%	P_2_
Total					0.220*			<0.001*
Health check-up controls	50,805	88.20	5,983	10.39		811	1.41	
Gastric cancer patients	528	85.30	52	8.40		39	6.30	
Sex					<0.001*			<0.001*
Female	21,034	85.53	3,139	12.76		419	1.70	
Male	30,299	90.11	2,896	8.61		431	1.28	
Age (years)					<0.001^#^			<0.001^#^
≤30	8,107	92.08	618	7.02		79	0.90	
31–40	12,123	89.69	1,252	9.26		142	1.05	
41–50	16,385	86.15	2,298	12.08		337	1.77	
51–60	9,689	85.71	1,420	12.56		196	1.73	
61–70	3,632	88.78	385	9.41		74	1.81	
>70	1,397	94.33	62	4.19		22	1.49	
Comorbidity					0.004^#^			<0.001^#^
None	46,868	88.35	5,441	10.26		738	1.39	
Non-digestive diseases	2,812	86.02	387	11.84		70	2.14	
Upper digestive tract benign comorbidities	1,653	86.91	207	10.88		42	2.21	
Anemia					0.835^#^			0.371^#^
None	50,342	88.18	5,916	10.36		830	1.45	
Mild	775	87.18	100	11.25		14	1.57	
Moderate-to-severe	216	89.63	19	7.88		6	2.49	

P_1_ and P_2_, compared to the “None” group; *chi-square test; ^#^Wilcoxon rank-sum test.

**Table 2:** Comparisons of serum CEA and CA19–9 according to family history of malignancies.

Risk factors	Family history of malignancies							
	None		Other malignancies			Gastric cancer		
	n	%	n	%	P_1_	n	%	P_2_
Serum CEA					0.336*			0.993*
Negative	50,188	88.16	5,912	10.38		831	1.46	
Positive	1,145	88.97	123	9.56		19	1.48	
Serum CA19-9					0.740*			0.639*
Negative	49,676	88.16	5,845	10.37		825	1.46	
Positive	1,657	88.51	190	10.15		25	1.34	
Serologic multiplex					0.093*			0.953*
CEA–/CA19-9–	48,694	88.16	5,730	10.37		808	1.46	
CEA–/CA19-9+	1,494	87.93	182	10.71		23	1.35	
CEA+/CA19-9–	982	88.15	115	10.32		17	1.53	
CEA+/CA19-9+	163	94.22	8	4.62		2	1.16	

IQR, interquartile range.

P_1_ and P_2_, compared to the “None” group; *chi-square test.

### Associations between risk factors and family history of gastric cancer

Univariate and multivariate analyses showed that anemia and the serologic multiplex were not associated with the presence of a family history of gastric cancer (**Table 3**). Moreover, persons with upper digestive tract benign comorbidities (aOR = 1.51, 95% CI 1.09–2.71) or those older than 40 years had a significantly higher risk of having a family history of gastric cancer (**Table 3**). Male participants had a significantly lower risk of having a family history compared to female participants (aOR = 0.72, 95% CI 0.63–0.82) (**Table 3**).

**Table 3:** Associations of family history of gastric cancer with baseline features and serology.

Risk factors	Univariate	Multivariate
	OR (95% CI)	aOR (95% CI)
Sex		
Female	Ref.	Ref.
Male	0.75 (0.65–0.86)	0.72 (0.63–0.82)
Age (years)		
≤30	Ref.	Ref.
31–40	1.17 (0.89–1.55)	1.19 (0.92–1.54)
41–50	1.99 (1.56–2.55)	2.02 (1.60–2.54)
51–60	1.95 (1.50–2.53)	2.00 (1.55–2.58)
61–70	2.03 (1.48–2.80)	2.07 (1.49–2.86)
>70	1.67 (1.03–2.68)	1.73 (1.10–2.71)
Comorbidity		
None or non-digestive diseases	Ref.	Ref.
Upper digestive tract benign comorbidities	1.55 (1.13–2.12)	1.51 (1.09–2.71)
Anemia		
None	Ref.	Ref.
Mild	1.08 (0.64–1.85)	0.94 (0.53–1.66)
Moderate-to-severe	1.73 (0.77–3.90)	1.47 (0.58–3.70)
Serologic multiplex		
CEA–/CA19-9–	Ref.	Ref.
CEA–/CA19-9+	0.92 (0.61–1.40)	0.84 (0.52–1.34)
CEA+/CA19-9–	1.04 (0.64–1.69)	1.00 (0.62–1.63)
CEA+/CA19-9+	0.79 (0.20–3.18)	0.67 (0.24–1.87)

CI, confidence interval; OR, odds ratio; Ref., reference.

### Associations between family history of gastric cancer and gastric cancer risk

Family history of gastric cancer was an independent risk factor for gastric cancer (aOR = 5.59, 95% CI 3.81–8.20), but family history of non-gastric malignancies was not (aOR = 1.10, 95% CI 0.73–1.38) (**Table 4**). Additionally, male sex (aOR = 1.78, 95% CI 1.43–2.20), age 41–50 years (aOR = 2.13, 95% CI 1.48–3.08), 51–60 years (aOR = 5.86, 95% CI 4.12–8.33), and >60 years (aOR = 17.34, 95% CI 12.26–24.53), upper digestive tract benign comorbidities (aOR = 9.65, 95% CI 7.59–12.27), anemia (aOR = 38.76, 95% CI 29.89–50.27), and serologic multiplex (aOR = 5.09, 95% CI 4.02–6.43) were significantly associated with an increased risk of gastric cancer (**Table 4**). The sensitivity analyses in subsets without and with a family history of gastric cancer showed consistent results (**Table 4**).

**Table 4:** Gastric cancer risks and subgroup analyses in subsets with or without a family history of gastric cancer.

Risk factors	All observations		Without family history		With family history	
	Univariate	Multivariate	Univariate	Multivariate	Univariate	Multivariate
	OR (95% CI)	aOR (95% CI)	OR (95% CI)	aOR (95% CI)	OR (95% CI)	aOR (95% CI)
Sex (male *vs.* female)	1.84 (1.54–2.19)	1.78 (1.43–2.20)	1.74 (1.45–2.08)	1.65 (1.33–2.04)	7.09 (2.75–18.31)	7.34 (0.23–235.40)
Age (years)						
≤40	Ref.	Ref.	Ref.	Ref.	Ref.	Ref.
41–50	2.87 (2.00–4.13)	2.13 (1.48–3.08)	2.75 (1.91–3.97)	2.12 (1.47–3.06)	Ref.	Ref.
51–60	7.80 (5.53–11.00)	5.86 (4.12–8.33)	7.21 (5.10–10.21)	5.57 (3.98–7.80)	7.86 (2.76–22.34)	8.53 (2.28–31.93)
>60	32.89 (23.73–45.59)	17.34 (12.26–24.53)	30.85 (22.23–42.83)	16.59 (11.90–23.13)	30.97 (11.34–84.57)	22.64 (6.22–82.41)
Comorbidity (upper digestive tract benign diseases *vs.* non-digestive diseases/none)	14.11 (11.80–16.86)	9.65 (7.59–12.27)	14.89 (12.41–17.87)	10.07 (8.07–12.56)	3.91 (1.54–9.94)	4.79 (1.42–16.17)
Anemia (present *vs.* none)	34.63 (29.01–41.34)	38.76 (29.89–50.27)	35.85 (29.91–42.99)	38.20 (29.90–48.81)	21.82 (8.41–56.61)	66.18 (2.04–2146.65)
Serologic multiplex (CEA+ and/or CA19-9+ *vs.* CEA–/CA19-9–)	9.46 (7.96–11.25)	5.09 (4.02–6.43)	9.72 (8.14–11.60)	5.06 (4.05–6.32)	7.07 (3.11–16.10)	6.05 (1.71–21.42)
Family history of cancer (*vs.* none)						
Other malignancies	0.84 (0.63–1.11)	1.01 (0.73–1.38)	N/A	N/A	N/A	N/A
Gastric cancer	4.63 (3.32–6.45)	5.59 (3.81–8.20)	N/A	N/A	N/A	N/A

aOR, adjusted odds ratio; CI, confidence interval; N/A, not applicable; OR, odds ratio; Ref., reference.

### Protocols of family history-driven endoscopic screening of gastric cancer

Compared to those without a family history of gastric cancer, persons with a family history of gastric cancer who were aged >40 years could be recommended for endoscopic screening (OR = 2.36, 95% CI 1.29–4.32; NNS = 75) (**Table 5**). In particular, a family history of gastric cancer in conjunction with age >60 years (OR = 8.90, 95% CI 3.84–20.61; NNS = 14), upper digestive tract benign comorbidities (OR = 16.78, 95% CI 7.03–40.06; NNS = 8), seropositivity of CEA/CA19-9 (OR = 20.77, 95% CI 9.15–47.14; NNS = 7), or anemia (OR = 80.11, 95% CI 33.07–194.05; NNS = 3) should be strong indications for endoscopic screening (**Table 5**). Therefore, the strength of the recommendations was stratified for family history-driven endoscopic screening (**Figure 1**).

**Table 5:** Decision-making protocol for family history-driven opportunistic endoscopic screening of gastric cancer.

Stepped sequential stratification	Health check-up controls		Gastric cancer patients		OR (95% CI)	NNS
	n	proportion (%)	n	detection rate (%)		
Absence of family history	56,787	98.99	580	1.01	Ref.	N/A
Family history alone	120	100	0	0	Omitted	N/A
Family history + male sex	91	100	0	0	Omitted	N/A
Family history + age 40–60 years	456	97.64	11	2.36	2.36 (1.29–4.32)	75
Family history + age >60 years	66	91.67	6	8.33	8.90 (3.84–20.61)	14
Family history + upper digestive tract benign comorbidities	35	85.37	6	14.63	16.78 (7.03–40.06)	8
Family history + seropositivity of CEA/CA19-9	33	82.50	7	17.50	20.77 (9.15–47.14)	7
Family history + anemia	11	55.00	9	45.00	80.11 (33.07–194.05)	3

CI, confidence interval; N/A, not applicable; NNS, number needed to screen; OR, odds ratio; Ref., reference.

**Figure 1:**
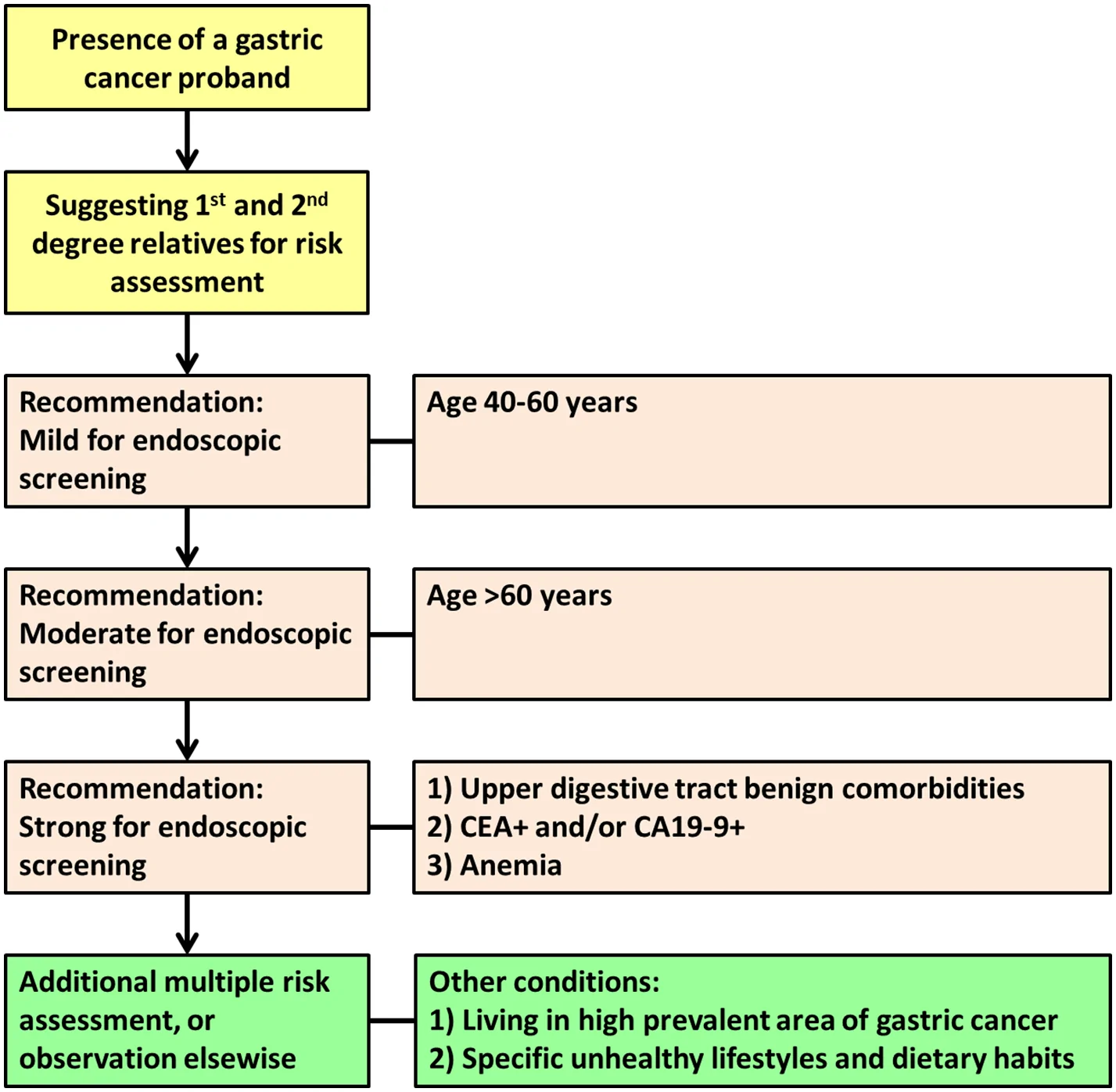
Flow chart for recommending endoscopic screening candidates according to family history of gastric cancer status in conjunction with related risk factors, in a reverse or forward manner.

### Cost-effectiveness of family history-driven protocols

Family history-driven endoscopic screening protocols had low detection rates and high FNRs (**Table 6**). The cost-effectiveness analysis ruled out protocol 1 (P1) in the decision-making approach because it did not improve the detection rate (**Table 6**, **Figure 2**). In the present study, no gastric cancer cases were found in the family history-associated subpopulation aged <40 years. The strong-recommendation protocol (P4) was preferred because it had the best cost-effectiveness. Moreover, based on willingness-to-pay considerations, risk stratification indicated that moderate-to-strong recommendations (P3) and mild-to-strong recommendations (P2) should be considered, with improved detection rates and considerable CER and ICER values. P2 might be considered given its optimal detection rate, regardless of direct medical costs. Additionally, the sensitivity analysis confirmed consistent decision-making results for P2 and P3 by varying the number of healthy check-up control numbers.

**Table 6:** Cost-effectiveness analysis of endoscopic screening protocols.

Screening protocol	Family history-driven candidates	Detection rate (per 1,000 persons)	FPR (%)	FNR (%)	Total direct medical cost (CNY)	CER	ICER	Decisions
P1	All candidates for direct endoscopy	0.67	1.4	93.7	495,550	739,741.8	1,232,098.4	Dominated
P2	≥mild recommendation	0.67	1.0	93.7	450,007	671,756.6	1,076,132.3	Willingness-to-pay
P3	≥moderate recommendation	0.48	0.2	95.5	177,746	369,572.0	407,293.1	Willingness-to-pay
P4	Strong recommendation	0.38	0.1	96.4	135,770	359,284.4	Ref.	Preferred

CER, cost-effectiveness ratio; FNR, false-negative rate; FPR, false-positive rate; ICER, incremental cost-effectiveness ratio; Ref., reference.

**Figure 2:**
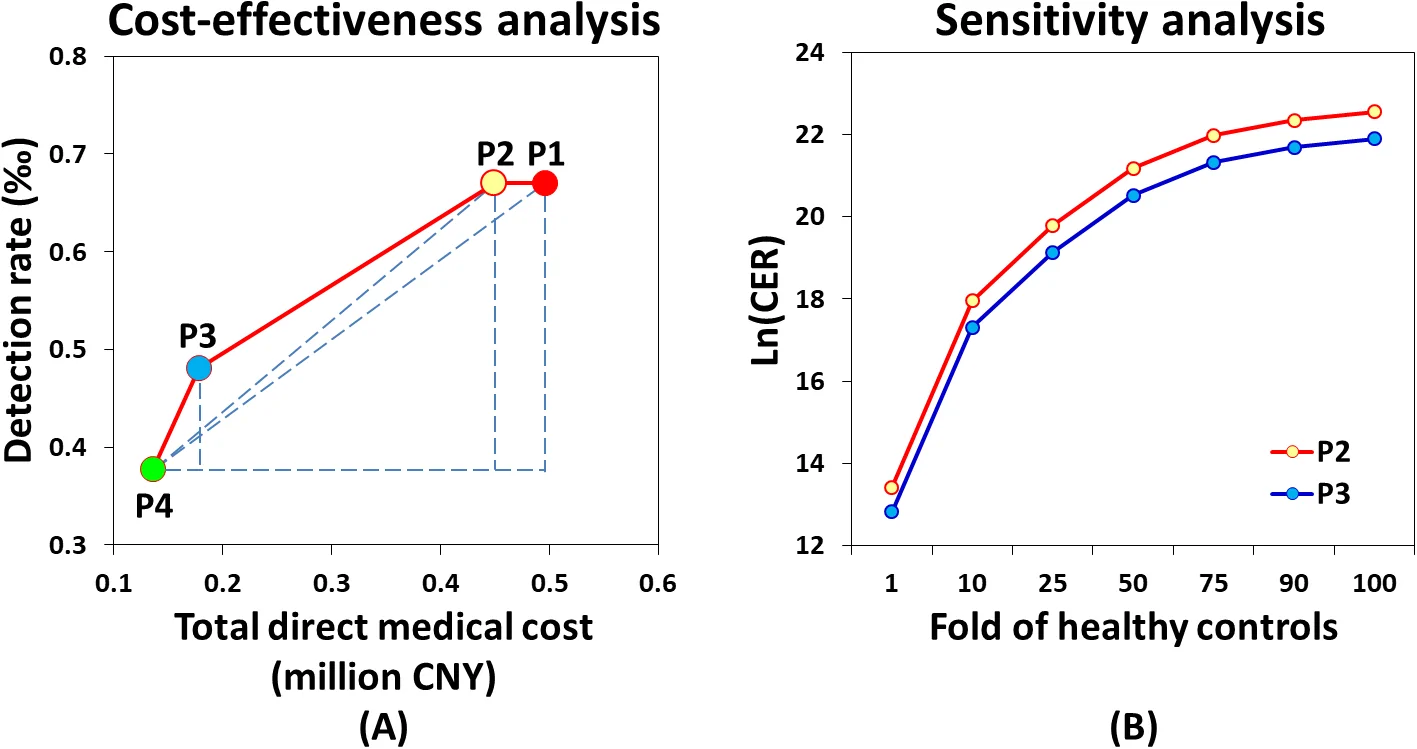
**(A)** Cost-effectiveness analysis of four protocols of family history-driven endoscopic screening (the vertical or horizontal dashed line represents the intercept, and the oblique dashed line represents the slope); **(B)** Sensitivity analysis by varying the number of healthy check-up control numbers. (CER, cost-effectiveness ratio; CNY, Chinese yuan).

## Discussion

This hospital-based large-scale cross-sectional study mainly showed that family history of gastric cancer was an independent risk factor for gastric cancer and was associated with an increased risk of upper digestive tract benign comorbidities. The decision-making protocol demonstrated that family history of gastric cancer, combined with other risk factors for gastric cancer, could increase detection rates of gastric cancer. For persons with a family history of gastric cancer who were older than 40 years, endoscopic screening should be mildly recommended in general practice. Endoscopic screening should be moderately recommended for those with a family history of gastric cancer if they were older than 60 years and strongly recommended in the presence of upper digestive tract benign comorbidities, seropositivity of CEA/CA19-9, or anemia. The present study optimizes the family history-driven strategy for recommending endoscopic screening candidates in both reverse and forward manners, which is expected to improve the efficiency of gastric cancer screening. However, the family history-driven strategy should not be applied alone but should be nested within comprehensive risk stratification for gastric cancer screening.

Currently, the incidence of gastric cancer in China remains high, while the overall case fatality rate is likewise elevated, primarily attributable to the low proportion of early-stage cases at diagnosis. Accordingly, the National Health Commission of the People’s Republic of China issued official guidelines for the early diagnosis and treatment of gastric cancer in 2024 (22). The criteria for defining high-risk individuals for gastric cancer screening include family history of gastric cancer, and second-degree relatives are incorporated as screening targets. However, in real-world practice, first- and second-degree relatives of a gastric cancer proband are insufficiently informed about the need to undergo gastric cancer screening; similarly, individuals with a family history of gastric cancer in the general health check-up population are inadequately advised to receive such screening. Moreover, even when recommendations have been communicated, the proportion of those who actually undergo standardized gastric cancer screening remains low, especially in rural areas (7). In other words, adherence to and implementation of reverse screening for gastric cancer based on family history require further strengthening. Based on practical experience, the strength of a physician’s recommendation may be correlated with the patient’s adherence to screening.

Therefore, the present study was conducted on the basis of the above rationale, with the aim of further stratifying the risk of gastric cancer among first- and second-degree relatives in families with a history of the disease. Such risk stratification may help physicians adopt a more decisive and firm stance when recommending reverse screening based on family history of gastric cancer. The present study does not contend that gastric cancer screening should rely solely on family history; rather, it represents a further partial optimization within the existing guideline framework, with a view to improving the cost-effectiveness of family-history-related reverse screening for gastric cancer.

The etiology of family history-associated gastric cancer is multifactorial, and the mechanism of carcinogenesis remains unclear (23). Familial clustering of gastric cancer may reflect shared genetic or environmental factors, or both (24). Relatives with a family history of gastric cancer have an increased incidence of precancerous gastric abnormalities and gastric cancer, which is confined to those with *Helicobacter pylori* infection (25, 26). Moreover, there is a synergistic effect of *Helicobacter pylori* infection and family history on the increased risk of gastric cancer (13, 27). Adult relatives (>20 years) with a family history of gastric cancer are recommended to undergo *Helicobacter pylori* screening and sequential eradication (24). Other carcinogenetic pathogens, such as the Epstein-Barr virus, hepatitis B virus, human papillomavirus, human cytomegalovirus, and John Cunningham virus, should also be investigated for familial aggregation (28–31). Early-onset gastric cancer patients may harbor mutations related to hereditary diffuse gastric cancer (24). Molecular investigations are suggested among early-onset gastric cancer patients, and identification of a proband with CDH1-mutated gastric cancer might enhance early detection and endoscopic screening within the affected family (23). In addition, harmful lifestyle factors such as smoking and alcohol consumption were more frequent in families with gastric cancer, while protective lifestyle factors such as physical activity were less frequent (32). Therefore, improving health awareness in families with a history of gastric cancer and evaluating and educating family behaviors may be effective preventive interventions (23).

In the largest study, which included 12 prospective cohorts with 550,508 participants in the Asia Cohort Consortium, a family history of gastric cancer increased the risk of gastric cancer in the Asian population, regardless of sex, tumor site, and histologic type (33). However, one study showed that a family history of gastric cancer was associated only with an increased risk of non-cardia cancer and not cardia cancer (34). The status of *Helicobacter pylori* infection might have been a confounder. Nevertheless, eradication of *Helicobacter pylori* can reduce the risk of gastric cancer among first-degree relatives with a family history of gastric cancer (35). In addition, the present study showed that a family history of non-gastric malignancies was not associated with the risk of developing gastric cancer, which is consistent with the result of another study (34). However, subgroup analysis in another study showed that a family history of other digestive system cancers or any early-onset cancer could increase gastric cancer risk (36), a finding that should be interpreted with caution.

As mentioned above, in the recent guidelines of China and relevant collaborations, a family history of gastric cancer in first- or second-degree relatives is defined as one of the high-risk factors for gastric cancer and is an indication for endoscopic screening among adults older than 40–45 years (16, 17). Generally, conventional endoscopic screening is no longer recommended for individuals aged >75 years, while magnetically controlled capsule gastroscopy can be considered as an alternative (16, 17). Recommendations in the Americas also recognize that relatives of gastric cancer patients have a higher risk of developing gastric cancer than persons without a family history of gastric cancer, but the upper age limit for endoscopic screening candidates is 65 years (24). Additionally, in Western countries, second-degree relatives with a family history of gastric cancer have not been recommended as a high-risk subpopulation for gastric cancer (37). Probably because of the relatively lower incidence of gastric cancer in western countries, universal screening is considered not to be cost-effective for the general population and would overuse limited endoscopic resources (37). However, several earlier studies that included second-degree relatives with a family history of gastric cancer showed consistent results indicating an increased risk of gastric cancer (38–41). China is an area with a high prevalence of gastric cancer, so a more intensive family history-driven strategy that recommends second-degree relatives with a family history of gastric cancer as endoscopic screening candidates may be reasonable.

Several limitations should be considered. First, this study recruited merely a hospital-based cross-sectional population, rather than a natural population. Sampling bias may be introduced to a certain extent. The proportion of gastric cancer was much higher than the prevalence of gastric cancer in a natural population. Therefore, the positive findings may have been amplified by the scale-up effect. Meanwhile, the number of participants with positive family history of gastric cancer was relatively small, which resulted in very wide confidence intervals and might introduce unstable estimates. Second, those health check-up controls did not obtain endoscopic confirmation of gastric cancer-free status. A minor risk of case misclassification might theoretically exist. Third, the interaction between family history of gastric cancer and serologic stratification combining serum *Helicobacter pylori* antibody, pepsinogen-I, pepsinogen-II, and gastrin-17 requires further investigation (42, 43). A more robust serologic protocol may provide additional information for selecting family history-driven endoscopic screening candidates. Fourth, patients with early-stage gastric cancer comprised only a small proportion (<20%) (6, 44), which may amplify the power of early detection. Fifth, the surveillance frequency for family history-driven endoscopic screening candidates remains uncertain and requires further cohort studies for clarification. A recent national guideline recommended surveillance at intervals of no longer than 5 years (17). However, a 2-year surveillance interval is recommended in certain guidelines instead (13).

Finally, some confounders, including smoking status and alcohol consumption, were not accounted for in the present study owing to the relatively high proportion of missing data in the gastric cancer group. *Helicobacter pylori* infection was classified as an upper digestive tract benign comorbidity during the initial data processing. The potential association between *Helicobacter pylori* infection and family history of gastric cancer can be separately investigated based on our hospital’s experience. Data on quality-adjusted life years (QALYs), life-years, downstream treatment costs, and complication-related outcomes are not available within the present cross-sectional study. If feasible, we plan to conduct a cohort study in future work to capture these outcome measures. The justification of the willingness-to-pay threshold depends on the budget affordability of public medical insurance agencies; therefore, it is difficult for the present study to propose recommendations for a specific threshold. In addition, the derivation of these graded-strength recommendations was not independently validated. Accordingly, these clinical recommendations should be treated as exploratory rather than definitive. The above findings require cautious confirmation through more robust investigations.

## Conclusions

Family history of gastric cancer is an independent risk factor for gastric cancer. Optimized risk stratification based on family history can improve the cost-effectiveness and efficiency of detecting gastric cancer. Based on willingness-to-pay considerations, family history-driven candidates aged over 40 years can be actively recommended for opportunistic endoscopic screening. The mechanism by which a family history increases gastric cancer risk and a more tailored family history-driven endoscopic screening strategy require further investigation.

## Data Availability

The datasets presented in this article are not readily available, while the in-house data will be available by emailing the corresponding author. Requests to access the datasets should be directed to X-ZC, chenxinzu@scu.edu.cn.
